# Effect modification by developmental stage of embryos on the association between late follicular phase progesterone elevation and live birth in fresh transfers

**DOI:** 10.1186/s12884-023-05342-w

**Published:** 2023-01-13

**Authors:** Fei Li, He Cai, Li Tian, Haiyan Bai, Juanzi Shi

**Affiliations:** 1 Assisted Reproduction Center, Northwest Women’s and Children’s Hospital, 73#, Houzaimen North Street, Xi’an City, Shaanxi Province China; 2 The Second Affiliated Hospital of Xi’an Medical University, Xi’an City, Shaanxi Province China

**Keywords:** Progesterone, Live birth, GnRH antagonist, Blastocyst, Cleavage

## Abstract

**Background:**

Late follicular phase progesterone elevation (LFPE) during ovarian stimulation is associated with reduced live birth rates (LBRs) after cleavage-stage embryo transfer. However, due to better synchronization with a stimulated endometrium, prior studies shown that LFPE had no effect on transferring embryos at blastocyst stage. The study aim to exam whether the developmental stage of embryos and serum progesterone levels on the day of human chorionic gonadotropin (hCG) administration jointly affect the odds of live birth in fresh fresh IVF/intracytoplasmic sperm injection (ICSI) cycles.

**Methods:**

The single-center retrospective cohort study included a total of 4,471 fresh embryo transfer cycles with 2,342 at cleavage stage versus 2,129 at blastocyst stage. Patients underwent IVF/ICSI with ovarian stimulation in gonadotropin-releasing hormone antagonist protocol. The serum progesterone level was examined both as a continuous variable and as a categorical variable by quartiles. Analysis was performed using the generalized estimating equations framework and multivariate regression models.

**Results:**

LBRs were inversely associated with progesterone as a continuous variable on the day of hCG in both the cleavage-stage (crude OR 0.87, 95%CI 0.73–1.03; adjusted OR 0.80, 95% CI 0.65–0.98) and the blastocyst-stage (crude OR 0.66, 95%CI 0.56–0,78; adjusted OR 0.61, 95%CI 0.50–0.73) groups. The interaction testing was highly significant (*P* = 0.018) indicating an effect modifying role of stage of embryos transferred on the association of pregesterone values with the LBRs in fresh cycles. A similar pattern for a greater reduction in ORs for live birth in cycles with blastocysts transfer was also observed when progesterone was analyzed by interquartile ranges. The findings remained unchanged in subgroup analysis stratified by types of ovarian response.

**Conclusions:**

In fresh cycles, detrimental effect of late follicular phase progesterone elevation on live birth was more prominent in blastocyst-stage group compared with that in clevaged-stage group.

**Supplementary Information:**

The online version contains supplementary material available at 10.1186/s12884-023-05342-w.

## Introduction

The association of late follicular phase progesterone elevation (LFPE) during controlled ovarian stimulation (COS) with the probability of pregnancy in fresh IVF/intracytoplasmic sperm injection (ICSI) cycles has been extensively studied for many years. Evidence has now convincingly demonstrated that LFPE is associated with lower live birth rates in fresh assisted reproductive technology (ART) cycles [1–5], which could explain for the adverse influence of progesterone rise on endometrial receptivity, subsequent embryo-endometrial asynchrony and altered gene expression during the window of implantation [6–8]; rather than an inferred impairment on oocyte or embryo quality [6, 9].

Among fresh cycles, women undergoing transfer of blastocyst-stage embryos had better results than those undergoing transfer of cleavage-stage embryos. Due to better synchronization with a stimulated endometrium, one would expect that extending the embryo culture to day 5 and transferring the embryo at the blastocyst stage would be preferable in terms of premature progesterone rise. Papanikolaou et al. [10] described that modest rises of progesterone in the follicular phase had detrimental effect on pregnancy outcomes in transferring the embryo at the cleavage stage, whereas, no such adverse impact was observed in the blastocyst-stage group. It was postulated that the endometrium could correct itself by day 5 or that a blastocyst was more robust and could compensate for the endometrium asynchrony. However, the potential protective effect of extending culture to blastocyst stage has been challenged in recent years [11–13]. Levi-Setti et al. [13] have shown that the adverse impact on pregnancy rates also extended to blastocysts with a serum progesterone level > 1.5 ng/ml on the day of human chorionic gonadotropin (hCG) (pregnancy rates per transfer were 39.6 and 40.1% in blastocysts and cleavage embryos cohorts). In a study of 2,555 blastocyst transfers, a similar reduction in pregnancy rates was seen with progesterone levels > 2 ng/ml; however, this study did not have a group of cleavage embryo transfer to use for direct comparison. Whether a blastocyst stage embryo transfer could ameliorate the adverse effect of LFPE has not been confirmed.

In addition to strategies alleviating the detrimental effects of the high progesterone, progesterone cutoff value is also a point of controversy. LFPE is usually taken as an elevation of serum P ≥ 1.5 ng/ml on the day of hCG triggering [14, 15]. However, the cut-off values were differ widely from 0.8 to 3.0 ng/ml [4]. It is problematic to define a specific threshold based on arbitrarily chosen values beyond which the progesterone levels would be considered abnormally high and an intervention would be reasonable [16]. The relationship between serum progesterone levels and pregnancy rates was non-linear [14], thus the use of receiver-operating characteristic (ROC) curve analysis may not be sufficient to predict success rates. Studies evaluating the role of progesterone as a continuous covariate in probability of live birth following fresh embryo transfer are warranted.

Given that the negative impact of pregesterone exposure was assumed to be primarily at the level of endometrium, we hypothesized that LFPE can impair fresh pregnancy outcomes, even in blastocysts transfer cycles [17, 18]. The primary objective was to check for a modifying effect of the developmental stage of embryos (cleavage-stage versus blastocyst-stage) on the association of serum progesterone concentrations with the probability of live birth.

## Materials and methods

### Study population

This was a non-interventional, retrospective, observational, single-centre cohort study. Patients undergoing autologous IVF/ICSI with gonadotropin-releasing hormone antagonist for ovarian stimulation (not in the context of a modified natural cycle) were enrolled from June 2018 to May 2021. This research was approved by the Ethics Committee of Northwest Women’s and Children’s Hospital (No. 2022007) and written informed consent was obtained from all participants.

Only patients who were underwent fresh embryo transfer were included in the analysis. The exclusion criteria were: 1) women aged > 42 years; 2) cycles with day 6 embryo transfer; 3) Cycles in which the serum progesterone concentrations were not measured on the day of hCG administration. A total of 4,471 IVF/ICSI with fresh embryo transfer cycles (2,342 in day 3 cleavage-stage and 2,129 in day 5 blastocyst-stage) were analyzed (Figure S1).

### Controlled ovarian stimulation and embryo transfer

Multifollicular ovarian stimulation was performed with gonadotropin-releasing hormone (GnRH) antagonist and mixed follicle stimulating hormone (FSH)/Lutenizing hormone (LH). The antagonist was initiated when the lead follicle was 14 mm in size. Ovarian stimulation was achieved by several types of FSH (Gonal-F®, Serono, Switzerland; Puregon®, N. V. Organon, Netherlands; Urofollitropin®, Livzon, China) or by FSH combined with LH activity (hMG®, Livzon, China). Dose adjustments were performed according to ovarian response as monitored by means of vaginal ultrasonographic scans and measurements of serum estradiol (E_2_). When more than two follicles reached 17 mm, oocyte maturation was triggered with 10 000 IU of hCG (Livzon, China) or with 250 mcg of recombinant hCG (Ovidrel®, Serono, Switzerland). Serum progesterone levels were obtained on the day of hCG trigger. Oocyte retrieval occurred 36 h later and insemination was achieved with conventional IVF or ICSI as clinically indicated. Sperm preparation, IVF and ICSI procedures, and embryo culture were carried out as described by Shi et al. [19].

A grading criterion was used to evaluate the quality of the cleavage-stage embryos [20]. Grade 1 and Grade 2 embryos with ≥ 6 symmetrical blastomeres of equal size were considered top-quality embryos. Generally, patients with two or more top-quality cleavage-stage embryos were likely to be candidates for blastocyst culture. Embryos with 6–8 cells on Day 3 and < 20% fragmentation were regarded as good-quality embryos (Grades 1 and 2) [21]. In the case of blastocysts, degree of expansion, inner cell mass and trophectoderm were graded according to a scoring system previously described [22]. In our practice, when the progesterone level had increased to more than 3.0 ng/ml before hCG, all embryos were frozen and transferred in sequential thawed cycles. All embryo transfers were performed using a Wallace catheter and guided with an abdominal ultrasound scan. One or two of the embryos were transferred into the uterus on day 3 or 5. Luteal support with progesterone supplementation was maintained to 10 weeks of gestation.

### Endocrine assays

Serum concentrations of progesterone, E_2_, FSH and LH were measured routinely prior to initiation (Day 2–4 of the cycle). Whole blood was collected between 7:30 and 10:00 am on the day of hCG administration for the immediate measurement of progesterone, E_2_ and LH.

Hormone assay was tested using a Beckman-Coulter Unicel DxI 800 Access Analyzer based on chemiluminescence and commercially available kits (Beckman-Coulter, USA). Analytical sensitivity was 0.1 ng/mL for progesterone, 20 pg/mL for E_2_, 0.2 mIU/mL for FSH and 0.2 mIU/mL for LH. Intra- and interassay coefficients of variation were: 8.2% and 7.9% for progesterone, 6.0% and 12.0% for E_2_, 3.6% and 5.1% for FSH, 4.3% and 5.4% for LH, respectively.

### Outcome measures

We evaluated the association between the progesterone values on the day of hCG administration and the live birth rates (LBRs) in different stages of embryo at transfer. Live birth was defined as a live born infant at 24 weeks gestation or later. Secondary outcomes included biochemical and clinical pregnancy. Serum β-hCG was measured 14 and 17 days after oocyte retrieval. An increase in serum human chorionic gonadotropin levels above 20 IU per liter indicated pregnancy. Clinical pregnancy was defined as one or more observed gestational sac or definitive clinical signs of pregnancy under ultrasonography at 7 weeks after embryo transfer.

### Statistical analysis

The distribution of continuous variables is described with the use of the medians and interquartile ranges (IQRs). Categorical variables are presented as proportions and percentages of the total. Comparison of continuous variables among groups was performed with the use of the Student’s t-test or Mann–Whitney U-test depending on the normality of the distribution, while the Fisher’s Exact test was used to compare categorical variables. Adjusted odds ratios (aORs) with 95 percent confidence intervals were calculated using a multivariate logistic regression analysis, the models were fitted with a generalized estimating equation (GEE) to account for patients who underwent multiple cycles [23]. Two models were undertaken: model I included parameters significantly related to pregeterone elevation: female age, peak E_2_ values, gonadotrophin dosage and the number of oocytes retrieved; In Model II, all variables that showed significance and were thought to be clinically relevant to live birth were encompassed and adjusted-for as covariates. Progesterone values were analyzed both as a continuous variable and a categorical variable stratified by IORs. 

GEE models were used to perform interaction testing to determine whether the effect of progesterone on live birth and pregnancy rate was similar in cleavage- and blastocyst- stage embryo transfers. The progesterone values were treated as a continuous variable in the GEE models to evaluate the effect of progesterone across its entire range. To check the robustness of the conclusions in different ovarian responders, these analyses were stratified by oocyte yield. Sensitivity analysis for restricting on the first cycle of IVF and/or ICSI treatment were performed. All *P*-values are two sided with a statistically significance level determined at *P* < 0.05. Data were analyzed with the statistical packages R (v.3.4.3; R Foundation for Statistical Computing, Vienna, Austria) and EmpowerStats (R) (X&Y Solutions, Inc., Boston, MA).

## Results

### Patient characteristics and serum progesterone values

A total of 4,471 fresh autologous ART cycles met inclusion criteria, including 2,342 cleavage-stage transfers and 2,129 blastocyst-stage transfers. The distribution of progesterone values on the day of hCG administration was shown in Figure S2. Statistical distribution of the progesterone levels was as follows: 25th percentile, 0.76 ng/mL; 50th percentile, 1.08 ng/mL; and 75th percentile, 1.45 ng/mL. Progesterone values on the day of hCG were statistically higher in blastocyst-stage cohort, although the median difference was small at 0.17 ng/ml (1.0 ng/ml and 1.17 ng/ml, respectively). This could be partially explained because patients undergoing transfer with blastocyst-stage embryos yield higher ovarian response and better prognosis: younger, lower FSH levels, fewer gonadotrophin usage, more oocytes retrieved and embryos available (Table 1). The characters of cleavage-stage group and blastocyst-stage group were different, however, our study was design to test whether progesterone elevation affected the two cohorts in a different manner, rather to determine whether reproductive outcomes were different in cleavage-stage and blastocyst-stage embryo transfer cycles.

**Table 1:** Baseline and stimulation characteristics of cleavage-stage and blastocyst-stage embryo transferred cohorts

	Cleavage-Stage Embryo Transferred	Blastocyst-Stage Embryo Transferred	*P*-value
	*N* = 2342	*N* = 2129	
Progesterone on day of trigger (ng/ml)	1.00 (0.69–1.37)	1.17 (0.84–1.53)	< 0.001
Year of treatment, n (%)			< 0.001
2018	272 (11.61%)	90 (4.23%)	
2019	677 (28.91%)	563 (26.44%)	
2020	786 (33.56%)	803 (37.72%)	
2021	607 (25.92%)	673 (31.61%)	
Female age (years)	33.00 (30.00–37.00)	32.00 (30.00–36.00)	< 0.001
Female age (years), n (%)			< 0.001
< 30	945 (40.35%)	977 (45.89%)	
30–34	461 (19.68%)	464 (21.79%)	
35–37	420 (17.93%)	374 (17.57%)	
38–42	516 (22.03%)	314 (14.75%)	
Male age (years)	34.00 (31.00–38.00)	33.00 (30.00–37.00)	< 0.001
BMI (kg/m^2^)	22.32 (20.31–24.84)	22.38 (20.40–24.61)	0.495
BMI (kg/m^2^), n (%)			0.813
< 20	1627 (69.47%)	1503 (70.60%)	
20–24.99	164 (7.00%)	156 (7.33%)	
25–29.99	462 (19.73%)	391 (18.37%)	
≥ 30	86 (3.67%)	77 (3.62%)	
Missing	3 (0.13%)	2 (0.09%)	
Smoking, n (%)	651 (27.80%)	611 (28.70%)	0.503
Infertility duration (years)	3.00 (2.00–5.00)	3.00 (2.00–5.00)	0.006
Gravidity,n (%)			0.202
0	922 (39.47%)	889 (41.99%)	
1	628 (26.88%)	533 (25.18%)	
≥ 2	786 (33.65%)	695 (32.83%)	
Parity,n (%)			0.119
0	1694 (72.67%)	1574 (74.63%)	
1	559 (23.98%)	454 (21.53%)	
≥ 2	78 (3.35%)	81 (3.84%)	
Cause of infertility, n (%)			< 0.001
Male factor	429 (18.32%)	343 (16.11%)	
Tubal factor	1257 (53.67%)	1314 (61.72%)	
Ovulatory disorder	328 (14.01%)	229 (10.76%)	
Endometriosis	146 (6.23%)	69 (3.24%)	
Unexplained	182 (7.77%)	174 (8.17%)	
Basal FSH (mIU/mL)	7.79 (6.34–9.85)	7.29 (6.04–9.01)	< 0.001
Antral follicle count	6.00 (5.00–9.00)	8.00 (6.00–11.00)	< 0.001
Rank of cycle	1.00 (1.00–2.00)	1.00 (1.00–1.00)	< 0.001
Fertilization methods, n (%)			< 0.001
IVF	1714 (73.19%)	1762 (82.76%)	
ICSI	621 (26.52%)	359 (16.86%)	
IVF + ICSI	7 (0.30%)	8 (0.38%)	
Gonadotrophins (IU)	2400.00 (2025.00–3000.00)	2400.00 (1875.00–2925.00)	< 0.001
Gonadotrophin type, n (%)			< 0.001
FSH	484 (20.67%)	544 (25.55%)	
FSH + LH	1858 (79.33%)	1585 (74.45%)	
LH on day of trigger (mIU/mL)	2.28 (1.42–3.72)	2.11 (1.27–3.36)	< 0.001
Estradiol on day of trigger (pg/ml)	1557.60 (991.00–2565.97)	2338.48 (1541.88–3540.00)	< 0.001
Oocytes retrieved	5.00 (3.00–8.00)	8.00 (5.00–10.00)	< 0.001
Two-pronuclei	3.00 (2.00–4.00)	5.00 (4.00–7.00)	< 0.001
Viable embryos	2.00 (1.00–3.00)	5.00 (4.00–7.00)	< 0.001
Number of embryos transferred	1.00 (1.00–2.00)	1.00 (1.00–1.00)	< 0.001
1, n (%)	1218 (52.01%)	1841 (86.47%)	< 0.001
2, n (%)	1117 (47.69%)	288 (13.53%)	
3, n (%)	7 (0.30%)	0 (0.00%)	

Data are expressed as median (interquartile range) or percentage of outcome

Serum progesterone values on the day of hCG administration were positively and significantly correlated with E_2_ and number of oocytes retrieved, total gonadotropin dosage, basal FSH, antral follicle count (*P* < 0.0001 for all). Progesterone levels were negatively correlated with patient age (coeficient of correlation -0.01; *P* < 0.001).

### Association of serum progesterone levels with pregnancy outcomes

**Fig. 1:**
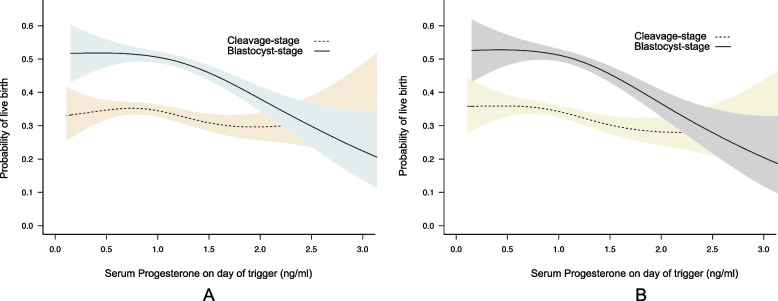
Smooth curve fitting of the effect of serum progesterone values on live birth in cleavage-stage and blastocyst-stage embryo transfers. Progesterone levels (ng/ml) are plotted on the x-axis, and live birth rates are on the y-axis. **A** crude model; **B** adjusted for covariates

Unadjusted and Multivariate GEE analysis showed that progesterone as a continuous variable was negatively associated with LBRs in both cohorts (crude OR 0.87, 95%CI 0.73–1.03; aOR 0.80, 95% CI 0.65–0.97) in cleavage-stage and (crude OR 0.66, 95%CI 0.56–0,78; aOR 0.59, 95%CI 0.49–0.72) in blastocyst-stage. The association of progesterone with the probability of live birth according to developmental stage of embryos was shown in Fig. 1. As concentrations of serum progesterone processively increased, biochemcial and clinical pregnancy rates were also decreased significantly in the two cohorts (Figures S3 and S4).

### Stage of embryos is a moderator of the effect of LFPE on live birth

The detrimental effect of LFPB was more prominent in the blastocyst-stage group (OR 0.51, 95% CI 0.40–0.66) compared with that in the cleavage-stage group (OR 0.75, 95% CI 0.66–0.85). The negative association of progesterone on both cohorts persisted in adjusted models controlling for confounding variables (Table 2). With the increase of progesterone values, there was a trend toward non-significance of embryo transfer day. In order to test for a moderating effect of the types of embryo staged on the effect of progesterone on LBRs, while controlling for important confounders, an interaction term was introduced in the multivariable GEE models models. Interaction testing between progesterone and developmental stage of embryos transferred for live birth was highly significant (*P* = 0.018), suggesting the effect of progesterone elevation on LBRs was more pronounced on blastocyst cohorts (Table 2).

**Table 2:** Effect modification of stage of embryos on influence of progesterone on probability of live birth

	Cleavage-Stage Embryo Transferred			Blastocyst-Stage Embryo Transferred			*P-value for interaction test		
	Crude	Model I^a^	Model II^b^	Crude	Model I^a^	Model II^b^	Crude	Model I^a^	Model II^b^
	OR (95% CI) *P*-value	aOR (95% CI) *P*-value	aOR (95% CI) *P*-value	OR (95% CI) *P*-value	aOR (95% CI) *P*-value	aOR (95% CI) *P*-value			
Progesterone as a continuous variable	0.87 (0.73, 1.03) 0.095	0.79 (0.65, 0.97) **0.021**	0.80 (0.65, 0.98) **0.032**	0.66 (0.56, 0.78) ** < 0.001**	0.59 (0.49, 0.72) ** < 0.001**	0.61 (0.50, 0.73) ** < 0.001**	**0.021**	**0.018**	**0.016**

Generalized estimate equation were used

*CI* Confidence interval, *OR* Odd ratio

^a^Model I model adjusted for: age, gonadotrophin consumption, basal FSH, AFC, E_2_ levels on trigger day, oocytes retrieved

^b^Model II adjusted for: age, gonadotrophin consumption, basal FSH, AFC, E_2_ levels on trigger day, oocytes retrieved, BMI categories, number of embryos viable and transferred

^*^P-value for interaction test: 2-way interaction of stage of embryo transfer (cleavage vs blastocyst) and serum progesterone concentrations on live birth

The relationship between stage of embryos at transfer and progesterone with LBRs was also analyzed by IQRs. Following blastocysts transfer, there was a significant difference in LBRs as a function of the serum progesterone level (< 0.76: 52.15%; 0.76–1.08: 49.90%; 1.08–1.45: 49.66%; and > 1.45: 39.53%) (Table 3). The difference in the live birth between the two cohorts was 18.29% when progesterone was < 0.76 ng/ml, 13.78% for the range 0.76–1.08, 16.65% for the range 1.08–1.45, and 10.14% when progesterone was > 1.45 ng/ml, demonstrating a trend for the consistent greater negative effect of elevated progesterone among patients with blastocyst transfers.

**Table 3:** GEE models evaluating the effect of progesterone as categorical variable (interquartile range) on probability of live birth

	Cleavage-Stage Embryo Transferred				Blastocyst-Stage Embryo Transferred			
	Live birth	Crude	Model I^a^	Model II^b^	Live birth	Crude	Model I^a^	Model II^b^
Progesterone (ng/ml)	N (%)	OR (95% CI) *P*-value	aOR (95% CI) *P-* value	aOR (95% CI) *P-* value	N (%)	OR (95% CI) *P-* value	aOR (95% CI) *P-* value	aOR (95%CI) *P-* value
Q1 (< 0.76)	235 (33.86%)	1.0	1.0	1.0	206 (52.15%)	1.0	1.0	1.0
Q2 (≥ 0.76- < 1.08)	225 (36.12%)	1.10 (0.88, 1.39) 0.393	1.03 (0.82, 1.31) 0.784	1.03 (0.81, 1.31) 0.796	258 (49.90%)	0.91 (0.70, 1.19) 0.501	0.92 (0.71, 1.21) 0.565	0.93 (0.71, 1.21) 0.576
Q3 (≥ 1.08- < 1.45)	171 (33.01%)	0.96 (0.76, 1.22) 0.755	0.92 (0.71, 1.19) 0.510	0.93 (0.72, 1.20) 0.563	289 (49.66%)	0.90 (0.70, 1.17) 0.448	0.87 (0.66, 1.14) 0.311	0.89 (0.68, 1.17) 0.396
Q4 (≥ 1.45)	149 (29.39%)	0.81 (0.63, 1.04) 0.101	0.71 (0.53, 0.94) **0.018**	0.72 (0.54, 0.97) **0.030**	251 (39.53%)	0.60 (0.46, 0.77) ** < 0.001**	0.54 (0.41, 0.71) ** < 0.001**	0.55 (0.42, 0.73) ** < 0.001**

Generalized estimate equation were used

*CI* Confidence interval. *OR* Odd ratio, *Q* Quartile

^a^Model I model adjusted for: female age, gonadotrophin consumption, basal FSH, AFC, E_2_ levels on trigger day, oocytes retrieved

^b^Model II adjusted for: female age, gonadotrophin consumption, basal FSH, AFC, E_2_ levels on trigger day,oocytes retrieved,BMI categories, number of embryo viable and transferred

#### Subgroup analysis

We further investigated the impact of elevated serum progesterone in subgroups according to types of ovarian response (low [< 5 oocytes], normal [5–15 oocytes], and high [> 15 oocytes]). A similar pattern for a greater reduction in ORs for live birth in cycles with blastocyst transfer could be observed across all ranges of ovarian response (Figure S5).

#### Sensitivity analysis

The finding remained unchanged in sensitivity analysis, when restricting on the first ART cycle. The greater negative effect of elevated serum progesterone levels on live birth was observed in blastocyst transfer cohort (Interaction testing, *P* = 0.010).

## Discussion

### Principal findings

The results of our study suggested a modifying effect of developmental stage of embryo (cleavage-stage versus blastocyst-stage) on the association between LFPE and the probability of live birth in women undergoing fresh GnRH antagonist co-treated cycles. Increased serum progesterone levels on the day of hCG administration indeed correlated with a decline in live birth, irrespective of the stage of embryos at transfer. Moreover, the negative impact of LFPE was more pronounced on the blastocyst-stage group than that on the cleavage-stage group. This finding was present when progesterone treated as a continuous variable (Interaction test, *P* = 0.018) and consistent in all IQR groups evaluated, although the interaction was not statistically significant.

To overcome the detrimental effect of LFPE, fresh blastocyst transfer as a clinical approach has been proposed. Prior work has suggested that the negative effect of premature progesterone elevation may be mitigated by transfer of blastocyst-stage embryos. An earlier study involving 482 patients suggested that increased progesterone levels do not correlate with decreased pregnancy outcomes in blastocyst transfer [10]. However, the lack of negative effect could be due to the study being underpowered (207 in the blastocyst transfer group and 51 with progesterone elevation). A systematic review with various progesterone elevation thresholds analyzed, including the study above shown that premature P elevation had a similar negative effect on pregnancy outcome in fresh cycles, independent of developmental stage of embryos at transfer [4]. Similarly, Hill et al. [3] demonstrated that elevated progesterone had a similar negative interaction across embryo stage and embryo quality, supporting that high good embryo characteristics could not compensate for the endometrium asynchrony. Agreement was present between the present and the previous large sample studies regarding the negative effect of LFBP in fresh LBRs, regardless with cleavage- or blastocyst- stage embryos transfer. However, the results of this study suggested that patients with blastocysts had a greater reduction in their odds of live birth compared to the patients with cleavage embryos transfer (interaction testing was highly significant). This means that for women with premature progesterone elevation, the expected difference in the probability of live birth between blastocyst- and clevage- stage embryo transfer is greatly reduced. The adverse effect of progesterone on pregnancy outcomes in fresh cycles was stronger in blastocysts transfer.

A number of studies have previously tried to evaluate but failed to detect a significant moderating effect of the days of embryo at transfer on the association between elevated progeserone levelss and the probability of pregnancy achievement [3, 12]. However, the results should be interpreted with caution due to the flaws in the data processing step, variable progesterone cutoffs (arbitrary or not) and different ovarian stimulation protocols. First, it should be noted that negative impact from progesterone elevation comes from subtle increase during ovarian stimulation. Thus, in order to provide a clear picture of the association of progesterone values and live birth after fresh embryo transfer, serum progesterone on the day of hCG were treated as a continuous covariate in our study. Furthermore, to evaluate the impact of progesterone on the different stage of embryo at tranfer, both the baseline live birth rate of the two groups should be evaluated. Consistent with literature, among the fresh cycles, the blastocyst transfer group had better results. However, with the progressively increased progesterone values (as a continuous variable), the impact of progesterone concentrations on live birth rates was different in the two seperate groups. With reduced statistical power, dichotomization could leads to type II errors, which were appropriately elaborated by Venetis and Tarlatzis [16, 24]. Thus, the significance of the interaction term observed currently might be attenuated by the previous researches for the inefficient methodological approach using certain cut-off progesterone point. Additionly, it has also been suggested that the probability of reduced pregnancy outcome after blastocyst transfers among patients of a subtle progesterone rise on the day of hCG administration may be dependent on the type of GnRH analogue used. An increase of progesterone before final oocyte maturation with hCG would lead to impaired LBRs after blastocyst transfer in GnRH agonist cycles but had no effect in GnRH antagonist cycles [2]. Lower incidences of premature progesterone rise in IVF with gonadotrophins and GnRH analogues have also been described in GnRH antagonist than in GnRH agonist cycles [4, 25, 26]. The differences between the analyses used in these studies may represent a possible reason for the discordance.

### Strengths and limitations

Unlike most previous studies, the current study we evaluated progesterone as both a continuous variable and categorical variable. Appropriate methodological approaches could increase the reliability of the results [27]. Another major strength of our study is in its real-world based data with a large sample size of women. All cases were treated at the same clinic; thus, use of the same quality-controlled progesterone assay throughout our study minimized the variability in the concentration data.

Our analysis also has some shortcomings. Firstly, this study is retrospective and the presence of residual unknown bias cannot be excluded. For this reason, the present analysis was adjusted for the most important patient and treatment characteristics in order to identify the potential association of the progesterone levels and likelihood of live birth. Secondly, the inclusion of varies cycles in which some patients contributed more than one cycle is a source of concern, although sensitivity analysis performed restricting patients in their first treatment cycle. Finally, the results from the current study might not be necessarily generalizable to patients undergoing natural cycle or stimulation with GnRH agonist.

### Biologic plausibility

The results supported the early studies that elevated progesterones at the end of at the end of stimulation causes faster advancement of the endometrial lining, which can lead to an endometrium to no longer be in the window of implantation when it comes time to transfer either a cleavage stage embryo or blastocyst. Saadat et al. [28] reported that the acceleration in endometrial maturation was persisted up until 7 days after the trigger, corresponding to the window of blastocyst implantation. A study by Van Voorhis et [29] found that blastocysts typically do not develop in vitro until day 5, whereas the mean age of in vivo matured blastocysts collected by uterine lavage was reported to be 4.5 days after ovulation [29, 30]. From a biological plausibility perspective, the effect of premature endometrium may be further exaggerated in the implantation potential of developmentally advanced embryos such as the blastocyst stage. In addition to the advanced endometrial development, evidence from a functional genomics analysis of endometrial receptivity found that high progesterone levels on the day of hCG administration profoundly alter the gene expression profile of the endometrial sampling 7 days after the trigger [8, 31]. Using microarray technology, Labarta et al. [8] detected that 13 out of the 25 key genes, which has been proved to be associated with endometrial receptivity were over-regulated in women with LFPE. Increased uterine Natural Killer (NK) cells in endometrium 7 days after hCG administration were also reported in cycles with premature elevated progesterone [32].

### Implications

Our analysis did not suggest any benefit from replacing cleavage-stage embryos with blastocyst-stage embryos for women with prematurely elevated progesterone at the end of stimulation. On the contrary, adverse pregesterone effect was further worsened for fresh cycles with blastocyst transfer. In this regard, blastocysts might be wasted in the fresh cycle in the presence of “high” progesterone. The decision to extend embryo culture to the blastocyst stage is routinely based on evaluation of embryo morphology. However, data in this study may aid clinicians to more precisely predict probability of live birth and stage of embryos at transfer tailored to individual value of progesterone at the day of hCG trigger. Similar to our analysis, the study published by Racca et al., [33] suggested that fresh transfer of the top-quality embryos in patients with elevated progesterone might have been the main cause of the decreased cumulative pregnancy outcomes. The safest strategy in the case of LFPE would be to apply to freeze-all and then transfer in a subsequent thawed embryos cycle when the hormonal effect on the endometrium will have faded [33, 34]. There is still an urgent need for providing solid evidence regarding the most effective way of managing women with premature progesterone on the day of hCG administration.

## Conclusions

In fresh IVF/ICSI cycles, the impact of LFPE on live birth was significantly modified by the stage of embryos at transfer. Adverse effect of progesterone rise on the day of trigger was more pronounced for transfer of blastocyst-stage embryos compared with transfer of clevaged-stage embryos. Future research should focus on the best approach to eliminate the hazards of LFPE for these patients during the current cycle and for future regimens.

## Supplementary Information

**Additional file 1:** **Figure S1.** Participants flowchart.

**Additional file 2:** **Figure S2.** Distribution of cases across ranges of serum progesterone levels (ng/ml) on the day of hCG administration.

**Additional file 3:** **Figure S3.** The effect of serum progesterone values on probability of biochemical pregnancy in cleavage-stage and blastocyst-stage embryo transfers. A.crude model; B. adjusted for covariates.

**Additional file 4:** **Figure S4.** The effect of serum progesterone values on probability of clinical pregnancy in cleavage-stage and blastocyst-stage embryo transfers. A.crude model; B. adjusted for covariates.

**Additional file 5:** **Figure S5.** The impact of elevated serum progesterone in subgroups according to types of ovarian response.

## Data Availability

The datasets used and/or analyzed during the current study are available from the corresponding authors on reasonable request**.**

## Abbreviations

LFPE: Late follicular phase progesterone elevation
ART: Assisted reproductive technology
LBRs: Live birth rates
hCG: Human chorionic gonadotropin
IVF: In vitro fertilization
ICSI: Intracytoplasmic sperm injection
COS: Controlled ovarian stimulation
ROC: Receiver-operating characteristic
GnRH: Gonadotropin-releasing hormone
FSH: Follicle stimulating hormone
LH: Lutenizing hormone
E_2_: Estradiol
IQRs: Interquartile ranges
aORs: Adjusted odds ratios
GEE: Generalized estimating equation generalized estimating equation;
NK: Natural Killer
