# Supplementary figures and images for: Effect modification by developmental stage of embryos on the association between late follicular phase progesterone elevation and live birth in fresh transfers
Source: BMC Pregnancy Childbirth. 2023 Jan 13;23:24. doi: 10.1186/s12884-023-05342-w (PMC9840276; doi:10.1186/s12884-023-05342-w)

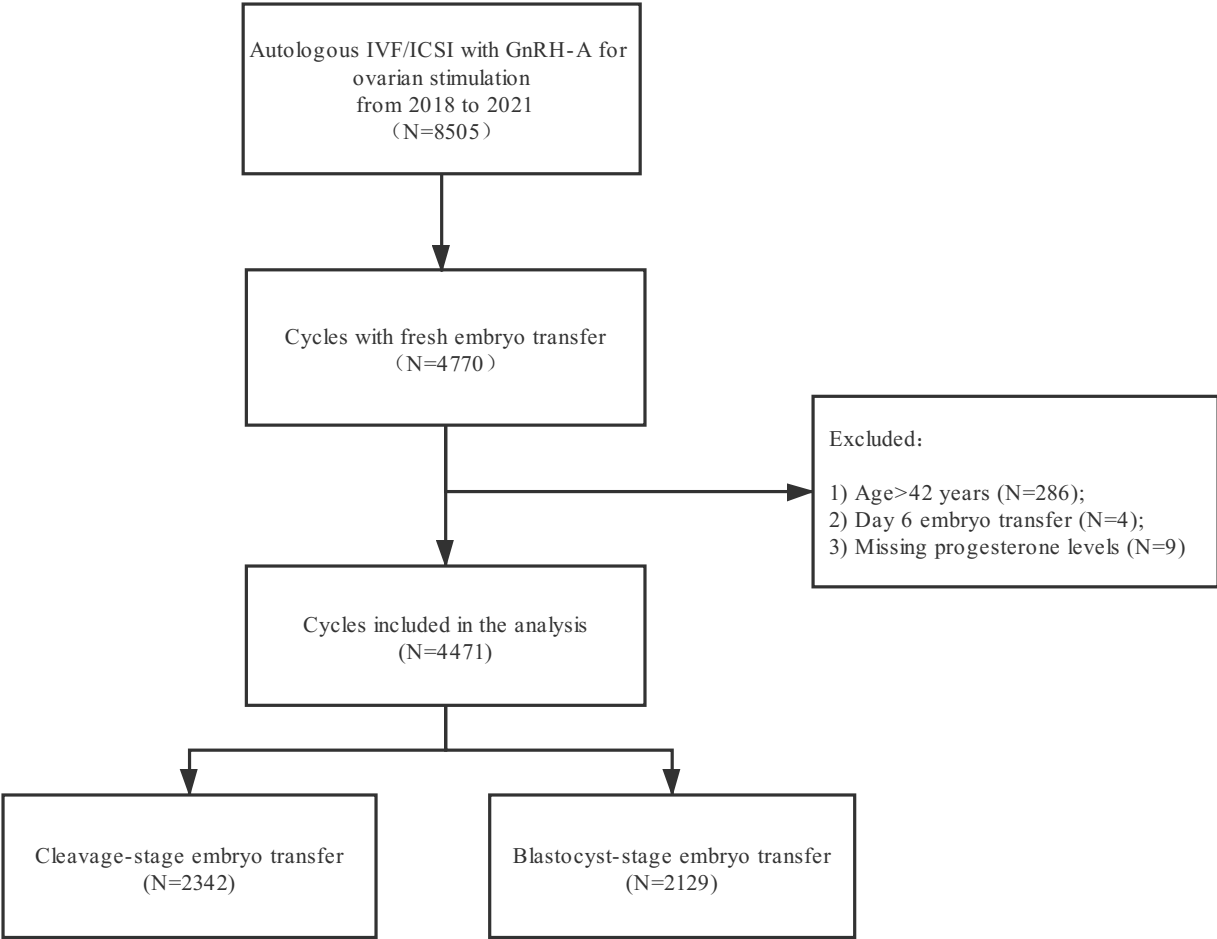

Figure S1. Participants flowchart.

Supplement: Supplementary file 1 — Additional file 1: Figure S1. Participants flowchart. [file 12884_2023_5342_MOESM1_ESM.pdf]
