# Supplementary material for: Effect modification by developmental stage of embryos on the association between late follicular phase progesterone elevation and live birth in fresh transfers
Source: BMC Pregnancy Childbirth. 2023 Jan 13;23:24. doi: 10.1186/s12884-023-05342-w (PMC9840276; doi:10.1186/s12884-023-05342-w)
Supplement: Supplementary file 3 — Additional file 3: Figure S3. The effect of serum progesterone values on probability of biochemical pregnancy in cleavage-stage and blastocyst-stage embryo transfers. A.crude model; B. adjusted for covariates. [file 12884_2023_5342_MOESM3_ESM.pdf]

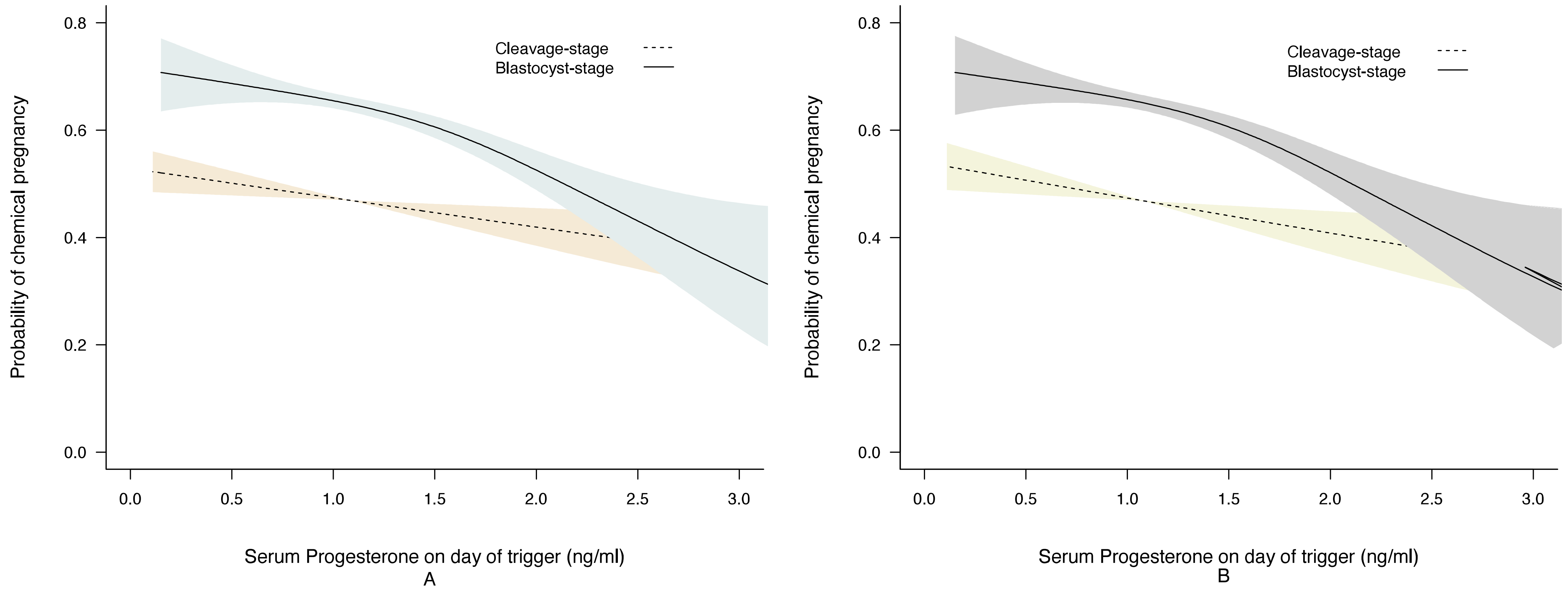

Figure S3. The effect of serum progesterone values on probability of biochemical pregnancy in cleavage-stage and blastocyst-stage embryo transfers. A.crude model; B. adjusted for covariates.
