# Supplementary material for: Effect modification by developmental stage of embryos on the association between late follicular phase progesterone elevation and live birth in fresh transfers
Source: BMC Pregnancy Childbirth. 2023 Jan 13;23:24. doi: 10.1186/s12884-023-05342-w (PMC9840276; doi:10.1186/s12884-023-05342-w)
Supplement: Supplementary file 5 — Additional file 5: Figure S5. The impact of elevated serum progesterone in subgroups according to types of ovarian response. [file 12884_2023_5342_MOESM5_ESM.pdf]

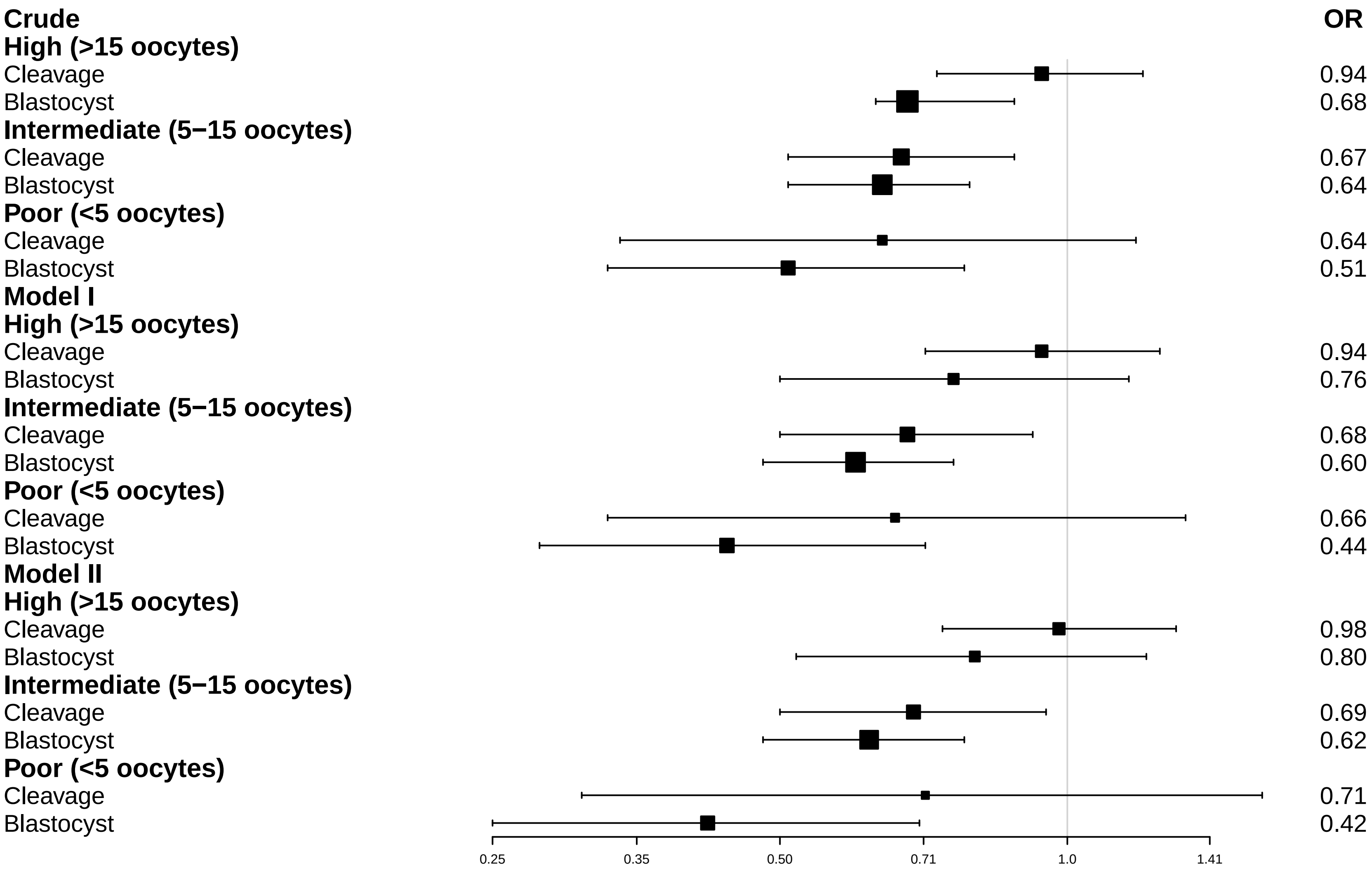

Figure S5. The impact of elevated serum progesterone in subgroups according to types of ovarian response.
